# Development of shuttle vector-based transformation systems for veterinary and zoonotic chlamydiae

**DOI:** 10.1128/spectrum.01641-25

**Published:** 2025-07-23

**Authors:** Nadja Fässler, Magdalena de Arriba, Michael Biggel, Martina Jelocnik, Nicole Borel, Hanna Marti

**Affiliations:** 1 Institute of Veterinary Pathology, Vetsuisse-Faculty, University of Zurich 634287, Zürich, Switzerland; 2 Institute of Food Safety and Hygiene, Vetsuisse-Faculty, University of Zurich 600627, Zürich, Switzerland; 3 School of Science, Technology and Engineering, University of the Sunshine Coast 276017, Sippy Downs, Queensland, Australia; 4 Center for Clinical Studies, Vetsuisse-Faculty, University of Zurich 30843, Zürich, Switzerland; Michigan State University, East Lansing, Michigan, USA

**Keywords:** *Chlamydiaceae*, *chlamydia*, plasmid, horizontal gene transfer, zoonoses, transformation rate, competence

## Abstract

In veterinary medicine, the obligate intracellular bacteria *Chlamydia* (*C.*) *abortus*, *Chlamydia caviae*, and *Chlamydia pecorum* are known to cause ovine enzootic abortion, conjunctivitis in guinea pigs, and ocular/urogenital disease in koalas, respectively. Studying the biology of these bacteria has been challenging due to a dearth of genetic tools. This study aimed to establish stable transformation systems for *C. abortus*, *C. pecorum*, and *C. caviae* by introducing shuttle vectors carrying green fluorescent proteins. With the aim to select the most suitable green fluorescent protein for the tracking of chlamydiae *in vitro*, we further compared the fluorescence intensity of GFP to that of mNeonGreen. Transformed shuttle vectors comprised the native plasmid of the chlamydial species of interest, an *Escherichia coli* origin of replication (*ori*), a beta-lactamase (*bla*) or spectinomycin (*aadA*) resistance gene, and GFP or mNeonGreen for heterologous fluorescence expression. We compared the success of a *C. suis*-tailored transformation protocol (Protocol A) to that of an alternative protocol for *C. psittaci* and *C. trachomatis* (Protocol B), both of which employ calcium chloride for competence induction. Stable transformants were obtained for *C. pecorum* and *C. caviae* using protocols A and B, respectively, and we found that the fluorescence intensity of heterologously expressed GFP is higher than that of mNeonGreen. In contrast, pre-incubation with trypsin-EDTA prior to the application of calcium chloride was needed to obtain transformants of *C. abortus*. In summary, we established protocols for stable calcium chloride-mediated transformation for *C. pecorum* and *C. abortus* and expanded upon the genetic toolbox of *C. caviae*.

**IMPORTANCE:**

Chlamydiae are a diverse group of bacteria impacting human and animal health. Many of the veterinary species, such as *Chlamydia abortus*, *Chlamydia caviae*, and *Chlamydia pecorum*, which cause reproductive disorders and/or conjunctivitis, are zoonotic pathogens leading to a potentially life-threatening disease in humans. Our understanding of these species has been hampered due to a lack of genetic tools. In this study, we developed calcium chloride-mediated transformation protocols for each of these species: chlamydiae are mixed with shuttle vectors containing the complete species-specific plasmid sequence, an *Escherichia coli* origin of replication, and an antibiotic resistance gene for selection. We could further show that certain chlamydial species become more susceptible to genetic modification if they are pre-treated with trypsin-EDTA prior to the addition of calcium chloride and the vector of interest. Overall, we demonstrate that species-specific protocol refinement is indispensable to render chlamydiae competent for genetic transformation.

## INTRODUCTION

Several species of the obligate intracellular bacterial family *Chlamydiaceae* found in livestock, pets, and wildlife harbor a zoonotic potential (1). In their animal hosts, the severity of the disease ranges from asymptomatic to mild conjunctivitis to life-threatening pneumonia and abortion depending on the pathogen involved and the affected host (1, 2). A well-known zoonotic *Chlamydia* species is *Chlamydia abortus*, which harbors avian and ruminant strains, of which the latter strains are responsible for enzootic abortion in sheep and goats (3, 4). Both strain groups may lead to community-acquired pneumonia (CAP) in humans (1, 5, 6), whereas ruminant *C. abortus* is most commonly involved in miscarriage in pregnant women (7). Other veterinary chlamydial species have also been identified as a cause of CAP, such as *C. caviae*, a conjunctivitis agent in guinea pigs (8). *C. pecorum* may possess a similar zoonotic potential (9). However, it is primarily considered a veterinary pathogen with a host range ranging from livestock to marsupials. In the koala, *C. pecorum* causes debilitating diseases, such as blindness and reproductive disorders, leading to a population decline in this protected species (1, 10). In livestock, the clinical impact of *C. pecorum* infections depends on the geographical region: while strains causing polyarthritis, encephalomyelitis, and abortion in ruminants have mostly been reported in Australia and New Zealand, the economic and clinical impacts of *C. pecorum* on livestock in Western Europe appear to be less severe (1, 10–12).

For these veterinary species, we relied mainly on gene-centric and/or whole-genome sequencing studies to increase our understanding of their virulence and evolution (13). Studies have shown that *C. pecorum* is phylogenetically diverse in terms of strain-dependent virulence (12, 14–18), with strains harboring a range of genes encoding for chlamydial virulence markers, including T3SS effectors, cytotoxins, and the chlamydial plasmid (13, 18, 19). Moreover, deletions in the native plasmid could potentially be responsible for the virulence of abortigenic *C. pecorum* sequence type 23 (15). The genetic diversity of *C. caviae* has only recently been investigated, revealing the circulation of few but distinct strains within the European guinea pig population (20, 21). One *in vitro* study identified an inclusion membrane protein encoded by incA and a T3SS effector (sinC) as potential virulence markers in *C. caviae* (22). Avian *C. abortus* strains carry a plasmid as well as a cytotoxin gene within the plasticity zone, which are both associated with virulence (4, 23). In contrast, ruminant *C. abortus* strains are highly conserved and have not revealed any distinct virulence groups (24). Overall, in-depth analysis of proposed virulence markers of these zoonotic veterinary chlamydiae has been hampered by a dearth of genetic tools. While such tools have recently been developed for the *Chlamydia* genus, most studies have focused on human chlamydiae. Consequently, most tools have been established for *C. trachomatis* and *C. pneumoniae*, as well as the animal model species *C. muridarum* (25, 26), while only a few are available for other chlamydiae, that is, *C. caviae* (22), *C. felis* (26), *C. psittaci* (27), and *C. suis* (28).

The overall low transformation efficiency in chlamydiae requires careful optimization (28, 29). For instance, one of the strongest factors for improving transformation rates in *C. suis* was a higher calcium chloride (CaCl_2_) concentration (100 µM) compared to the normally applied 50 µM (28). Here, we aimed to establish transformation systems for *C. abortus* and *C. pecorum* as well as to expand the genetic toolbox of *C. caviae*. We employed shuttle vectors that carry the complete sequence of their corresponding native plasmid as well as genes for heterologous fluorescence expression, similar to the first stable transformation protocol described for *C. trachomatis* (30). We first used a protocol optimized for *C. suis*, in which chlamydiae and shuttle vectors were incubated in 100 µM CaCl_2_ prior to infection of trypsinized cells (Protocol A [28]). Alternatively, a general protocol successfully used for *C. trachomatis* (30), *C. psittaci* (27), *C. pneumoniae*, *C. felis* (26), and TargeTron gene-knockouts in *C. caviae* (22) was employed (Protocol B). Further optimization was performed if both protocols failed to yield transformants.

## RESULTS AND DISCUSSION

### *C. pecorum* is stably transformed with Protocol A (100 mM CaCl_2_ for 1 h without cell co-incubation)

**Fig 1:**
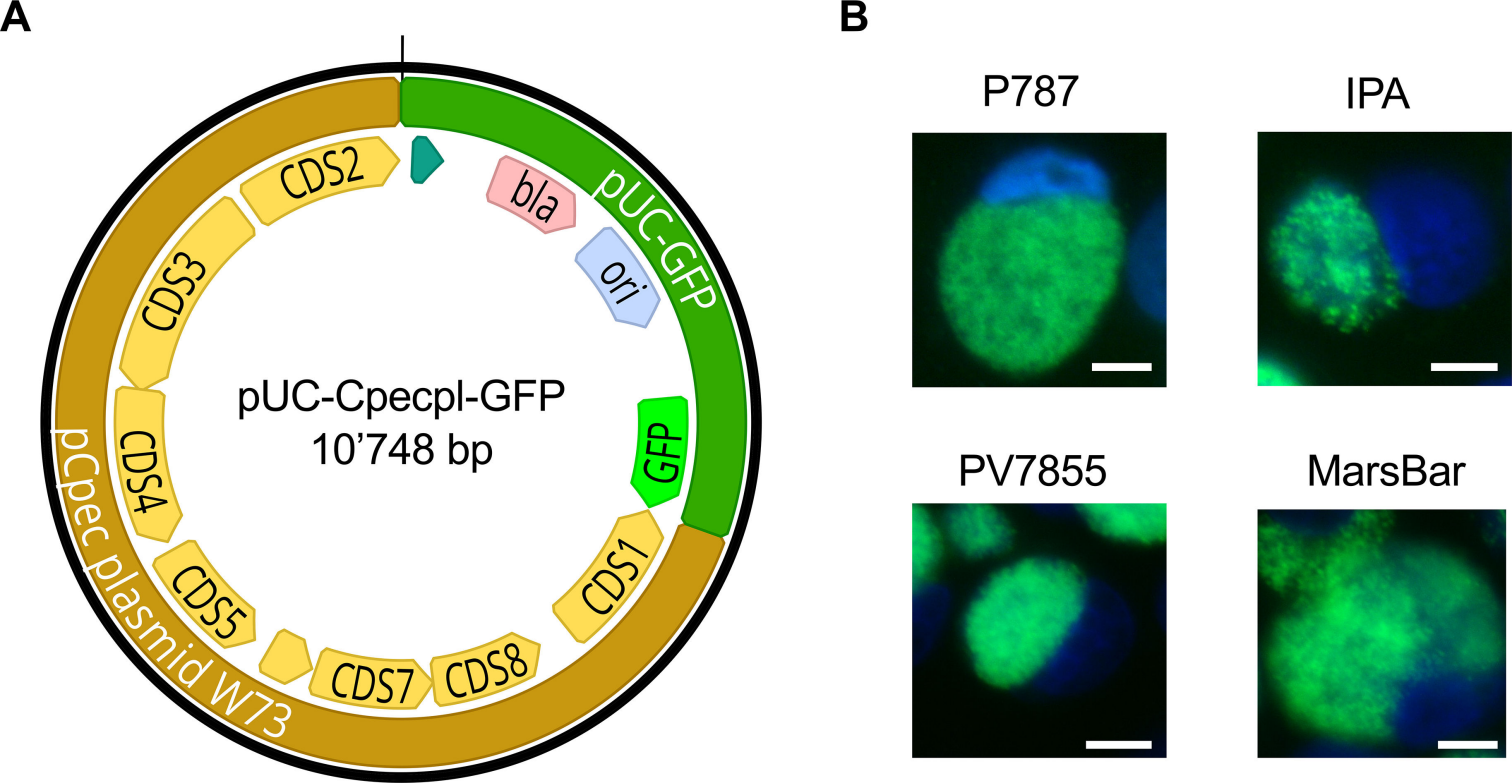
Transformation of *C. pecorum* strains with shuttle vector pUC-Cpecpl-GFP. (**A**) Map of the shuttle vector pUC-Cpecpl-GFP comprising the whole plasmid of *C. pecorum* strain W73 (labeled in light brown; coding sequences CDS 1–8 labeled in yellow) and pUC-GFP consisting of a beta-lactamase (bla, pale red), the pUC origin of replication (ori, pale blue), and GFP (neon-green). Image was created with Geneious version 2025.0 by Biomatters. Available from https://www.geneious.com. (**B**) Representative epifluorescence microscopy images of *C. pecorum* ovine P787 and IPA, chamois PV7855, and koala MC/MarsBar_2018 strains successfully transformed with pUC-Cpecpl-GFP at the time of collection after three to four passages. Images for DAPI (blue) and GFP (green, FITC channel) were taken individually using a 60× objective, and then merged. The white size bar represents 20 µm.

We first transformed *C. pecorum* using Protocol A (28), in which chlamydiae and vector are co-incubated for 1 h in 100 mM CaCl_2_ at room temperature prior to infection of trypsinized cells. Our shuttle vector, termed pUC-Cpecpl-GFP (Fig. 1A; Table S1), comprises the whole-plasmid sequence of *C. pecorum* strain W73 and expresses ampicillin resistance as well as a GFP fluorescence protein derived from pBOMB4R by Bauler and Hackstadt (31).

Transformation experiments were repeated three times or until transformants were obtained. With this strategy, we successfully transformed ovine *C. pecorum* strains P787 and IPA, PV7855 from a chamois, and koala strain MC/MarsBar_2018 but not porcine 1710S (Table 1; Fig. 1B). First transformants were observed after the second passage, similar to *C. suis* (28). The strain identity for each transformant was successfully confirmed by ompA sequencing (Table S2A). Each transformant stably maintained its shuttle vector for up to five passages in the presence and absence of ampicillin (Table S2B). In 100 counted inclusions per strain and condition, we detected no more than two GFP-negative inclusions, regardless of the passage number or the presence/absence of selective antibiotics (Table S2B). These results suggest that all transformants stably maintained the pUC-Cpecpl-GFP shuttle vector, including strain P787, a strain that naturally lacks a native chlamydial plasmid (32, 33). We expected this outcome based on our vector design, which mimicked the design of shuttle vectors for the stable transformation of *C. trachomatis* (30), *C. muridarum* (34), *C. suis* (28), *C. pneumoniae* (26), and *C. psittaci* (27). Plasmid-free strains of *C. trachomatis* (35), *C. muridarum* (34), and *C. pneumoniae* (26) were included in some of these studies and yielded similar results. Vector stability is likely conferred by the genes responsible for plasmid maintenance (36), namely, pgp 1, 2, 6, and 8 corresponding to coding sequences (CDS) 3, 4, 8, and 2, respectively (34). The importance of these CDS for plasmid stability was demonstrated by the development of vectors that contained only the chlamydial ori, in which successful transformants lost their vectors in the absence of antibiotic selection within the first three passages (37). Transformation of *C. pecorum* was attempted in another study using Protocol B with an incubation period of 30 min in 50 mM CaCl_2_ at room temperature, followed by 20 min of incubation with trypsinized cells before centrifugation and incubation (26). In that study, a shuttle vector designed for *C. pneumoniae* was used, but attempts to transform *C. pecorum* remained unsuccessful (26) possibly due to species barriers based on CDS 2 (*pgp 8*) as found for other chlamydial species (38). We also used Protocol B for the transformation of strain 1710S with pUC-Cpecpl-GFP. However, these attempts remained unsuccessful (data not shown), indicating that Protocol B does not yield a higher transformation rate for *C. pecorum* than Protocol A.

**TABLE 1:** List of strains used in this study

Species	Strain	Host	Disease	Plasmid	Reference
*C. pecorum*	P787	Sheep	Polyarthritis	No	(32, 33)
*C. pecorum*	MC/MarsBar_2018	Koala	Chronic cystitis	Yes	(13, 19)
*C. pecorum*	IPA	Sheep	Polyarthritis	Yes	(39)
*C. pecorum*	PV7855	Chamois	Pneumonia	Yes	(32)
*C. pecorum*	1710S	Pig	Inapparent intestinal infection	No	(39)
*C. caviae*	GPIC	Guinea pig	Conjunctivitis	Yes	(22)
*C. abortus*	S26/3	Sheep	Abortion	No	(40)
*C. abortus*	15-70d24	Avian	No information	Yes	(41)

### GFP-carrying shuttle vectors yield transformants with higher fluorescence intensities than shuttle vectors with mNeonGreen carrying the same promoter

**Fig 2:**
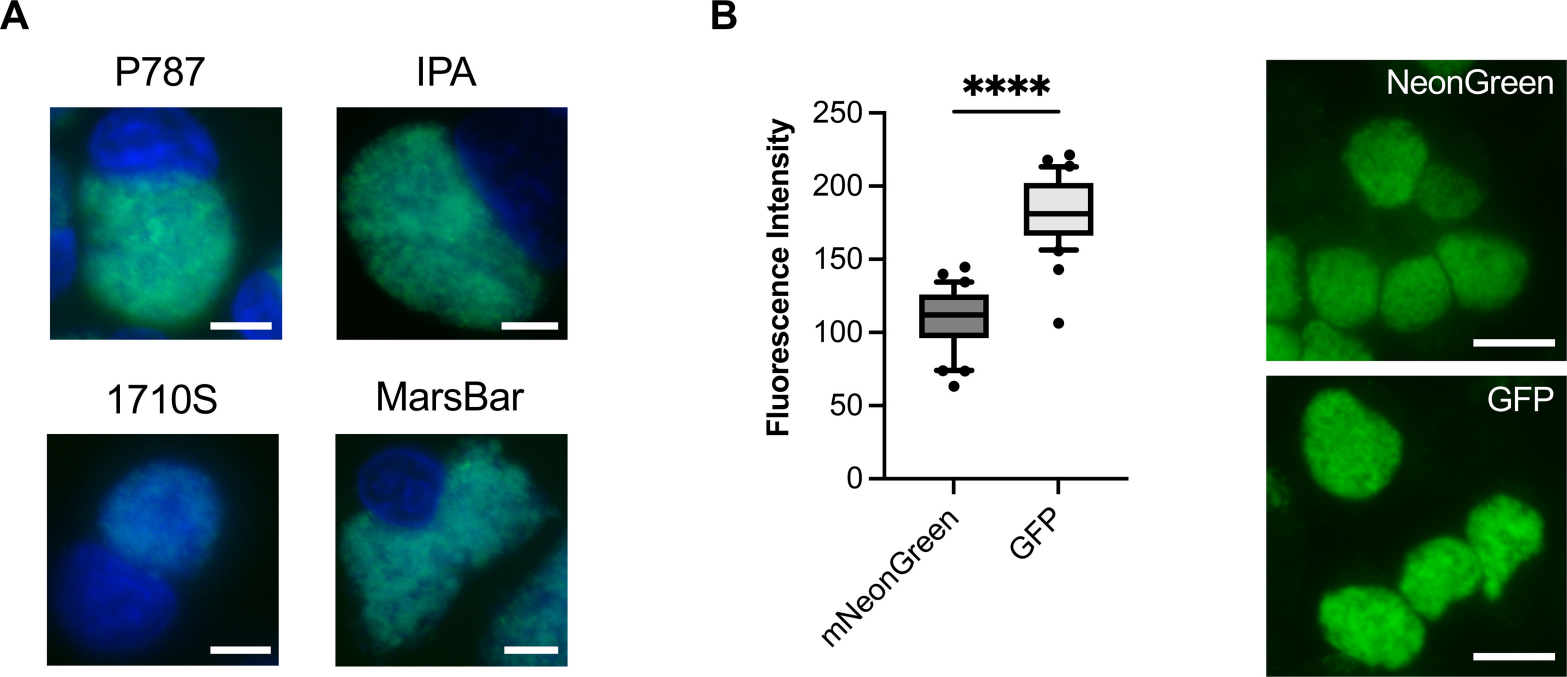
GFP-carrying shuttle vectors yield transformants with higher fluorescence intensities than shuttle vectors with mNeonGreen carrying the same promoter. (**A**) Representative epifluorescence microscopy images of *C. pecorum* strains transformed with a shuttle vector containing the *C. pecorum* W73 whole-plasmid sequence and mNeonGreen. Images for DAPI (blue) and mNeonGreen (green, FITC channel) were taken individually using a 60× objective and merged. The white size bar represents 20 µm. (**B**) The mean fluorescence intensity of 30 *C*. *pecorum* inclusions expressing NeonGreen was compared to that of GFP using strain P787. A Mann-Whitney test was used for the statistical analysis. Four asterisks (****) represent *P*-values < 0.0001. Representative images for each fluorophore were taken with a 40× objective (top right: GFP, bottom right: mNeonGreen). The white size bar represents 50 µm.

The fluorophore mNeonGreen is expected to express brighter green fluorescence and is considered to be less sensitive to photobleaching than GFP (42), making it an ideal candidate for longer exposure times. Consequently, in addition to pUC-Cpecpl-GFP, we designed pUC-Cpecpl-NeonGreen, in which GFP was replaced with mNeonGreen, using the same promoter (Table S1). We used *C. pecorum* strains P787, IPA, 1710S, and MC/MarsBar_2018 for transformation and were able to transform all strains with pUC-Cpecpl-NeonGreen (Fig. 2A). The mean fluorescence intensity of 1710S expressing mNeonGreen was significantly lower than that of other mNeonGreen-positive strains (Fig. S1). Considering the overall challenge to successfully transform 1710S, and that this strain does not naturally carry a plasmid (39), these data suggest that factors other than the plasmid sequence play a role in the plasmid maintenance of chlamydiae. For example, the weak fluorescence could indicate a lower number of plasmid copies in the transformed 1710S compared to other strains. However, since plasmid copy numbers in chlamydiae depend on various factors, including the chlamydial developmental cycle (43), comparing differences between strains and species remains difficult. Suboptimal growth conditions for 1710S could also affect their low transformation rate. A previous study looking into *in vitro* growth characteristics between different Chlamydia strains, including 1710S, showed that CaCo2 cells yield more inclusions with the same inoculum than monkey kidney cell lines, such as Vero cells (40), and possibly LLC-MK2 cells. Overall, these data demonstrate that transformation protocols should be further optimized for all chlamydial species (28) and that 1710S could be used as a model strain to investigate plasmid maintenance dynamics in *C. pecorum*.

Moreover, we observed that the mNeonGreen green fluorescence of all *C. pecorum* strains was notably weaker than for transformants expressing GFP, which stands in contrast to what we expected from their expression if introduced into mammalian cells (42). We confirmed this observation by measuring the mean fluorescence intensity of mNeonGreen-expressing P787 inclusions to that of GFP (Fig. 2B). These findings show that fluorophores for heterologous expression in chlamydiae need to be carefully considered and possibly compared for optimal use in infection experiments.

### Protocol B (50 mM CaCl_2_, 30 min, with cell incubation), but not Protocol A, yields stable *C. caviae* transformants

**Fig 3:**
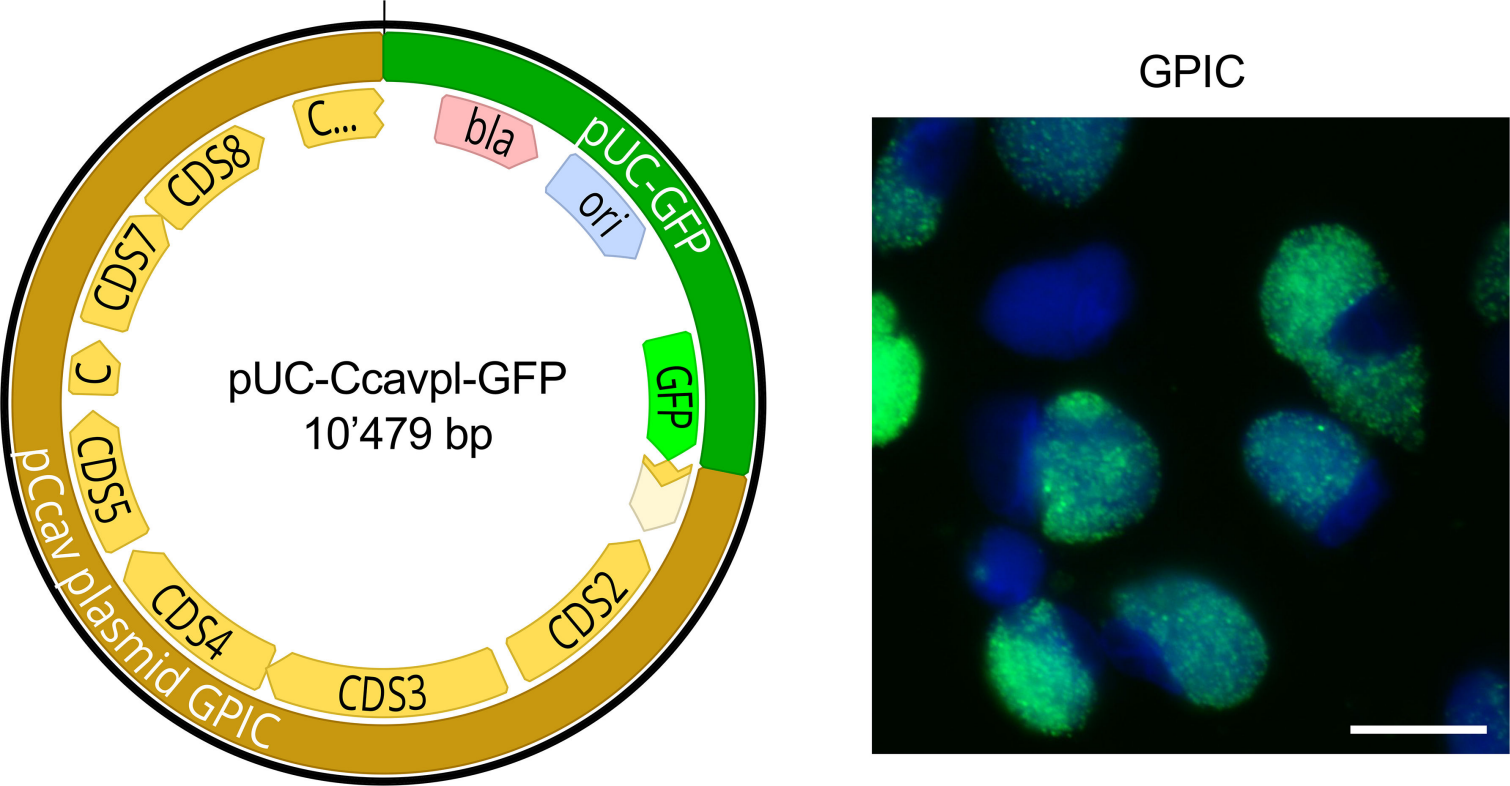
Transformation of *C. caviae* with shuttle vector pUC-Ccavpl-GFP. The left panel displays the vector map of the shuttle vector pUC-Ccavpl-GFP comprising the whole plasmid of *C. caviae* strain GPIC (labeled in light brown; coding sequences CDS 1-8 labeled in yellow). The shuttle vector possesses pUC-GFP, which contains a beta-lactamase (bla, pale red), the pUC origin of replication (ori, pale blue), and GFP (neon green). The image was created with Geneious version 2025.0 by Biomatters. Available from https://www.geneious.com. The right panel shows a representative epifluorescence microscopy image of successfully transformed *C. caviae* strain GPIC. Images for DAPI (blue) and GFP (green, FITC channel) were taken individually using a 60× objective and merged. The white size bar represents 50 µm.

During initial trials to transform *C. caviae* with Protocol A, we observed that 0.5 µg/mL ampicillin added at 6 h post-infection failed to sufficiently reduce infectivity for subsequent passages (data not shown). Consequently, we increased the ampicillin concentration to 5 µg/mL and employed both protocols A and B in parallel. We successfully transformed shuttle vector pUC-Ccavpl-GFP (Table S1) into *C. caviae* strain GPIC by incubation for 30 min in 50 mM CaCl_2_ at room temperature, followed by co-incubation with trypsinized cells for 20 min (Protocol B, Fig. 3). Transformation rates appeared to be higher than those of *C. pecorum* with GFP-positive inclusions already observed after the first passage and remained stable over at least five passages both in the presence and absence of antibiotic selection (Table S2B).

Interestingly, despite a comparatively high transformation efficiency under Protocol B, experiments with Protocol A remained unsuccessful for *C. caviae*. An alternative protocol with a 30 min incubation in 100 mM CaCl_2_, followed by cell co-incubation for 20 min, also remained unsuccessful (Table S2C). These findings demonstrate that an increase in the CaCl_2_ concentration does not necessarily increase the transformation efficiency, which is indicative of a complex interplay between individual factors that contribute to the successful transformation of different chlamydial species.

With the successful transformation of *C. caviae* using a shuttle vector, we expand upon the existing genetic toolbox of this species (22). Our GFP-expressing strain could be used in guinea pig infection models to better understand the dissemination of chlamydiae in an infected host over time. In the past, guinea pig models have not only been used to mimic genital *C. trachomatis* infections (44) but have also been used as a conjunctivitis model to investigate trachoma-like infections (45).

### Pre-incubation of *C. abortus* in trypsin-EDTA yields stable transformants, regardless of the transformation protocol

The same transformation strategy as for *C. caviae* was also applied to a ruminant (S26/3) and an avian (15-70d24) *C. abortus* strain (Table 1) using shuttle vector pUC-Cabpl-GFP comprising the whole-plasmid sequence of strain 15-70d24 (Fig. S1). However, all attempts to transform these strains remained unsuccessful, regardless of the protocol (Table S2C). In a previous study, we showed that the choice of antibiotic selection may lead to different transformation rates (28). Spectinomycin has become the standard selection for *C. trachomatis* in current protocols (46). Thus, in order to obtain a shuttle vector expressing spectinomycin rather than ampicillin resistance, we replaced the pUC-GFP backbone of pUC-Cabpl-GFP with the backbone of pSUmC-4.0 (46). The resulting shuttle vector, pUC-Cabpl-GFP_aadA, contains a spectinomycin resistance gene (aadA) and GFP (Table S1). Unfortunately, neither protocol A nor B was successful, suggesting a systemic problem for competence induction in *C. abortus*.

**Fig 4:**
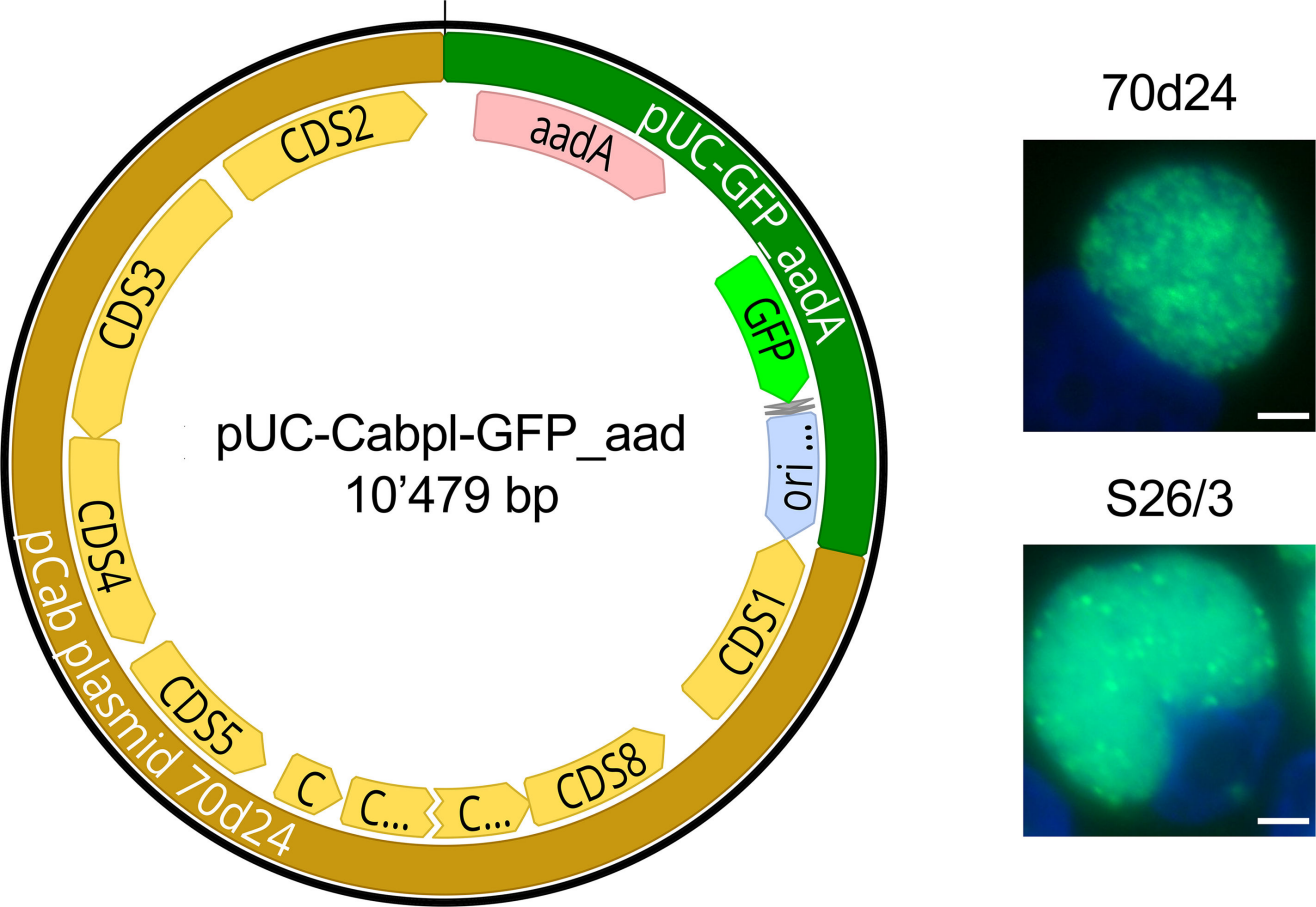
Transformation of *C. abortus* with the shuttle vector pUC-Cabpl-GFP_aadA. The top panel shows the shuttle vector map of pUC-Cabpl-GFP_aadA comprising the whole plasmid of *C. abortus* strain 15-70d24 (labeled in light brown; coding sequences CDS 1-8 labeled in yellow) and the aadA-GFP backbone (dark green) comprising spectinomycin resistance (aadA, pale red), an ori (pale blue), and GFP (neon-green). The image was created with Geneious version 2025.0 by Biomatters. Available from https://www.geneious.com. The bottom panel displays representative epifluorescence microscopy images of successfully transformed *C. abortus* strains 15-70d24 (avian, top) and S26/3 (ruminant, bottom). Images for DAPI (blue) and GFP (green, FITC channel) were taken individually using a 100× oil objective and merged. The white size bar represents 10 µm.

In a previous study, we showed that trypsinization of cells strongly increased transformation rates of *C. suis* (28), suggesting that trypsin-EDTA pre-treatment could improve the transformation efficiency of chlamydiae as was shown for *Bacillus subtilis* (47), a bacterium with a potentially similar competence machinery as *Chlamydia* (48). Consequently, we pre-incubated *C. abortus* strains 15-70d24 or S26/3 with 0.1% trypsin-EDTA for 30 min prior to application of protocol A or B and obtained stable transformants for both strains (Fig. 4). Both protocols yielded transformants for 15-70d24, but Protocol A was more reliable, especially if a minor change was applied: instead of mixing chlamydiae/vector with trypsinized cells after the 1 h incubation period, they were inoculated onto confluent monolayers. Ruminant strain S26/3 was only successful if the vector concentration was increased from 7.5 to 75 µg (10-fold), and the amount of chlamydiae was increased from 1.0 × 10^8^ to 2.0 × 10^8^ inclusion-forming units (IFU, twofold). Both strains maintained shuttle vector pUC-Cabpl-GFP_aadA over at least five passages in the presence and absence of spectinomycin (Table S2B).

Whole-genome analyses of ruminant *C. abortus* have revealed that these strains fall into a highly conserved clade and form a distinct cluster that does not undergo homologous recombination (24). This genomic inertia could explain the difficulty in transformation, especially considering that ruminant *C. abortus* strains do not carry a natural plasmid in contrast to the recently characterized avian strains (4, 49, 50). The effect of trypsin-EDTA on *Chlamydia* has been studied, revealing that it causes a partial degradation of the major outer membrane protein (MOMP) (51) and reduces the adherence of elementary bodies to the cell (52, 53). The partial degradation of MOMP could contribute to the increased transformation efficiency of *C. abortus*, but additional studies are necessary to confirm this hypothesis.

### If present, the native plasmid is replaced by the shuttle vector upon transformation

All transformants were confirmed by Nanopore whole-genome sequencing to ensure the identity of all transformants generated in this study (Table S3). In addition, we screened for the presence of a native plasmid to confirm that the shuttle vector entirely replaced the native plasmid, as has been observed previously (28, 30). Our results confirmed the strain identity of all transformants and further showed that, when present in the original strain, the native plasmid was entirely replaced by the shuttle vector, as expected. Circular shuttle vector assemblies were obtained for all transformants, except for *C. caviae*, which had a fragmented assembly. To confirm its identity, the vector was re-transformed into *E. coli* and fully sequenced as described previously (28). Using this method, we confirmed that the vector used for transformation was identical to the vector that could be retrieved from GFP-expressing *C. caviae* (Table S3).

### Conclusions

Overall, we were successful in the establishment of stable transformation systems for three veterinary chlamydial species of zoonotic importance using species-specific shuttle vectors. Similar to previous studies, we found that, albeit successful, the transformation rates are very low for all species, with few to no visible transformants after the first two passages (28, 29). Of note, *C. caviae* appeared to yield more transformants than *C. pecorum*, while the transformation of *C. abortus* could only be induced with an additional trypsin-EDTA pre-incubation step, as well as a higher infectious dose and vector concentration for ruminant strain S26/3. Future optimization experiments may improve transformation success as previously shown for *C. suis* (28). Potential applications of our transformants could be in studies examining dissemination of *C. caviae* in a guinea pig infection model and *C. pecorum* and *C. abortus* dissemination in a sheep infection model, as well as the analysis of chlamydial virulence markers for each of these important pathogens.

## MATERIALS AND METHODS

### Media and cell culture

Experiments were performed with LLC-MK2 cells (rhesus monkey kidney cell line kindly provided by IZSLER, Brescia, Italy) cultured at 37°C with 5% CO_2_ as previously described (54) with few modifications (28). Briefly, we used antibiotic-free growth media consisting of minimal essential medium with Earle’s salts, 25 mM HEPES, L-glutamine-free (Gibco, Invitrogen, Carlsbad, CA, USA), 10% fetal calf serum (FCS, BioConcept, Allschwil, Switzerland), 2 mM GlutaMAX (200 mM, Gibco), and 0.4 g D-(+)-glucose (Sigma-Aldrich, St. Louis, MO, USA). Infection medium contained fivefold higher glucose concentrations and was supplemented with cycloheximide (1.5 µg/mL, Sigma-Aldrich), if needed. Sucrose phosphate glutamate (SPG) buffer with 218 mM sucrose (Sigma-Aldrich), 3.76 mM KH_2_PO_4_ (Sigma-Aldrich), 7.1 mM K_2_HPO_4_, and 5 mM GlutaMAX (GIBCO) was used for the long-term storage of *Chlamydia* stocks as described (55).

### *Chlamydia* stocks

For this study, the *C. pecorum* strains were generously provided by the Center for Bioinnovation, University of the Sunshine Coast, Sippy Downs, Australia (strains MC/MarsBar_2018 and IPA) and by Istituto Zooprofilattico Sperimentale della Lombardia e dell'Emilia Romagna, IZSLER, Italy (strain PV7855) or were generous gifts from J. Storz, Baton Rouge, LA, USA (strains W73, P787, and 1710S). The *C. caviae* strain was kindly provided by Dr. Barbara Sixt of Umea University, Sweden and originated from Roger Rank (University of Arkansas for Medical Sciences). The avian *C. abortus* strain 15-70d24 was kindly provided by Dr. Monika Szymanska-Czerwinska (Department of Cattle and Sheep Diseases, National Veterinary Research Institute, Pulawy, Poland), and the ruminant *C. abortus* strain was a gift from Dr. Jones (Moredun Research Institute, Edinburgh, UK) (Table 1).

Stocks of semi-purified *Chlamydia* were prepared according to established protocols (56). Briefly, chlamydiae were grown for up to five passages, aiming for 75–100 % infection in T75 flasks (TPP, Trasadingen, Switzerland). Lysis of infected cells was achieved by scraping them into the supernatant and mechanical disruption through vigorous vortexing with sterile glass beads (5 mm diameter, Sigma-Aldrich) for 1 min. Cellular debris was removed by centrifugation (500 × *g*, 4°C, 10 min), and the chlamydiae were pelleted with 10,000 × *g* at 4°C for 45 min. Resuspension was performed in SPG, aiming for a stock concentration ranging from 10^9^ to 10^10^ inclusion forming units per mL (IFU/mL). Aliquots were then stored at –80°C until use.

The exact stock concentration was expressed as IFU/mL and determined by subpassage titration performed in duplicate as described (28). In brief, SPG stocks were inoculated onto 96-well plates (TPP), followed by a threefold serial dilution. After centrifugation (1,000 × *g*, 1 h, 25°C), inocula were replaced with infection medium containing cycloheximide and incubated at 37°C for 48 h. Monolayers were then fixed in ice-cold methanol for 10 min, and an immunofluorescence assay (IFA) was performed to visualize chlamydial inclusions using the protocol described by Leonard et al. (57). In brief, cells were blocked for 30 min in phosphate-buffered saline (PBS, Gibco) supplemented with 1% bovine serum albumin (BSA, Sigma-Aldrich) after methanol fixation. Chlamydial inclusions were then labeled with a *Chlamydiaceae*-specific primary antibody diluted 1:200 in blocking solution (*Chlamydiaceae* LPS, Clone ACI-P; Progen, Germany). After a washing step in PBS, DNA was stained with 1 µg/mL 4′, 6-diamidino-2′-phenylindole dihydrochloride (DAPI, Molecular Probes, Eugene, OR, USA), and inclusions were visualized for 45 min with the secondary antibody Alexa Fluor 488 goat anti-mouse (Molecular Probes) diluted in blocking solution (1:500). Inclusion numbers were then counted in a whole well using the Nikon Ti Eclipse epifluorescence microscope (Nikon, Tokyo, Japan), which was used to determine IFU/mL.

### Shuttle vector construction and vector stock preparation

The detailed protocol was published and first described in a previous study (28) (https://dx.doi.org/10.17504/protocols.io.kxygxy53wl8j/v1; Chapters 1–3). Briefly, *Chlamydia* shuttle vectors were designed to comprise an origin of replication (ori) of *E. coli* (pUC) together with a fluorescent gene (mNeonGreen, GFP), an ampicillin resistance gene (bla), and the entire native plasmid sequence of the chlamydial species of interest. For *C. pecorum*, the native plasmid sequence was obtained from strain W73 (40, 58). For *C. abortus* and *C. caviae*, avian strain 15-70d24 and strain GPIC were used, respectively (Table 1). Primers for all shuttle vectors, exact composition, and vectors used for shuttle construction are listed in Table S1. Vector construction was performed by PCR amplification with overlapping primers using Phusion Hot Start II DNA Polymerase Kit (Thermo Fisher Scientific, Waltham, MA, USA), and assembly was performed with the HiFi DNA Assembly Cloning Kit (New England Biolabs, NEB; Ipswich, MA, USA). Assembled vectors were then transformed into NEB5alpha *E. coli*. Vector sequences were confirmed by whole-plasmid sequencing using the Full PlasmidSeq service based on the long-read sequencing technology performed by Microsynth AG (Balgach, Switzerland). Unmethylated vector DNA for chlamydial transformation was then produced in dam-/dcm- *E. coli* (NEB) as recommended (59). Vectors and fluorescent genes used for construction included the following plasmids: pL0M-S-mNeonGreen-EC18153, a gift from Julian Hibberd (Addgene plasmid #137075) (60), as well as pBOMB4R and pSUmC-4.0, which were kindly provided by Ted Hackstadt from MT, USA (31) and Kenneth A. Fields from KY, USA (46), respectively.

### Transformation of *Chlamydia*

Transformation was attempted with two different protocols. Both protocols are published (https://dx.doi.org/10.17504/protocols.io.kxygxy53wl8j/v1, Chapter 4). Protocol A was optimized for *C. suis* (28). Briefly, 6.25 × 10^6^ IFU of *Chlamydia* was mixed with 7.5 µg of shuttle vector in 100 µL of calcium chloride (CaCl_2_) solution containing 100 mM CaCl_2_ (Merck) and 20 mM Tris (Roche, Basel, Switzerland), with a pH adjusted to 7.4. Incubation was performed at room temperature for 1 h with mild agitation, followed by the addition of freshly trypsinized cells resuspended in 100 mM CaCl_2_ and seeding onto six-well plates. After centrifugation (1,000 × *g*, 35°C, 1 h), cultures were incubated for 6 h before addition of 1.5 µg/mL cycloheximide and 0.5 µg/mL ampicillin, unless stated otherwise (Thermo Scientific). Up to four passages were performed every 36–96 h, with possible positive cultures appearing after two passages. For the collection of successful transformants, samples were scraped into SPG and frozen at −80°C until further processing. A fresh coverslip seeded to confluence in a 24-well plate was infected with a 1:100 to 1:200 dilution of the culture for imaging of positive cultures. After centrifugation (1,000 × *g*, 25°C, 1 h) and an incubation period of 48 h, cultures were fixed with ice-cold methanol for 10 min. Coverslips were washed with PBS, stained with DAPI (1 µg/mL) for 10 min, and transferred to glass slides with FluoreGuard mounting medium (Hard Set; ScyTek Laboratories, Inc., Logan, UT, USA). Imaging was performed as described in the section “Fluorescence microscopy and imaging.”

If three attempts with this protocol remained unsuccessful, transformation was attempted with Protocol B, as previously described (26, 27, 30), with few modifications. Briefly, 6.25 × 10^6^ IFU of *Chlamydia* mixed with 15 µg of shuttle vector were incubated for 30 min at room temperature in 50 mM instead of 100 mM CaCl_2_. Cells were trypsinized and suspended in the same concentration of CaCl_2_. These suspensions were then mixed together and incubated for another 20 min at room temperature before they were processed as described above.

For *C. abortus*, the following protocol was used: 1 × 10^8^ (strain 15-70d24) or 2 × 10^8^ (S26/3) was incubated in 0.1% trypsin-EDTA (Gibco) diluted in water for 30 min at room temperature. Subsequently, they were mixed with 7.5 (15-70d24) or 75 µg (S26/3) and processed according to Protocol A, unless stated otherwise. Following the 1 h incubation period, strains 15-70d24 and S26/3 were inoculated onto confluent monolayers of three and six wells of a six-well plate, respectively. Spectinomycin selection was 200 µg/mL at 6 h post-infection, 300 µg/mL for the first passage, and 400 µg/mL for the second passage; subsequent passages were performed 200–500 µg/mL depending on the infection rate.

### Initial strain identification by ompA sequencing

To ensure the identity of each strain before further processing, the near full-length ompA sequence was analyzed (the detailed protocol is published in: Chapter 5, protocols.io; https://dx.doi.org/10.17504/protocols.io.kxygxy53wl8j/v1). In brief, genomic DNA was extracted from transformant cultures using the Qiagen Blood & Tissue Kit (Qiagen, Hilden, Germany) according to the manufacturer’s instructions. The gene ompA encoding for MOMP was amplified by PCR, followed by Sanger sequencing at Microsynth AG. For *C. pecorum*, a species-specific full-length ompA PCR was used (39) as previously described (12). For *C. abortus* and *C. caviae*, an in-house PCR with the more general ompA primer pair BGP4 (5′-ATGAAAAAACTCTTGAAATCGG-3′)/CT90UF (5′-ACTGTAACTGCGTATTTGTCTG-3′) (61) was applied (62). Sequences were then compared against the National Center for Biotechnology Information databank with the Nucleotide Basic Local Alignment Search Tool (BLASTn) (https://blast.ncbi.nlm.nih.gov/Blast.cgi).

### Recovery of clonal stocks

Clonal purification was performed with a protocol derived from published work (59). Briefly, a 96-well plate (TPP) was divided into four quadrants. Two quadrants per plate were used per strain. The plate was seeded to confluence with LLC-MK2 cells, and the first well of a quadrant was inoculated with 10 µl of undiluted transformant stock. The first well of the second quadrant was inoculated with 1:100 diluted transformant stock. In both quadrants, a threefold serial dilution was first performed vertically in the first column, followed by horizontal serial dilution, resulting in 24 individually diluted wells per quadrant and 48 wells in total. After centrifugation (1,000 × *g*, 33°C, 1 h), inocula were replaced with infection medium supplemented with 5 µg/mL ampicillin or 300 µg/mL spectinomycin depending on the shuttle vector. After an incubation period of 48 h at 37°C, 96-well plates were investigated with a Nikon Ti Eclipse epifluorescence microscope (Nikon), and individual wells were selected that contained 0–1 visible fluorescent inclusions. These wells were scraped and passaged two times in 24-well plates (TPP) and one time in six-well plates (TPP) in selective antibiotics aiming for only a few inclusions, with infection rates of 50–90 and 75–100% in passages 1, 2, and 3. Stocks were then scraped into SPG and stored, and their concentration was determined as described above. The detailed protocol is published (https://dx.doi.org/10.17504/protocols.io.kxygxy53wl8j/v1, Chapter 6).

### Stability assay

Clonally purified stocks were inoculated onto 13 mm glass coverslips (Thermo Fisher Scientific) in 24-well plates seeded with LLC-MK2 cells aiming for a multiplicity of infection (MOI) of 0.1 and passaged five times every 48–72 h by scraping the wells and using 1:500 of the culture to infect fresh cells both in the presence and absence of 5 µg/mL ampicillin or 300 µg/mL spectinomycin depending on the shuttle vector. After initial infection (P0) and at the fifth passage (P5), coverslips were fixed in methanol, stained with DAPI, and mounted as described above in the section “Transformation of *Chlamydia*.” Two coverslips per condition (with or without selection) were semi-quantitatively assessed by counting 100 inclusions either at 400 or 1,000× magnification with a Leica DMLB fluorescence microscope. Each inclusion was identified by DAPI and the presence or absence of the appropriate fluorophore. Next, the number of fluorescent inclusions was expressed as a percentage of total inclusions.

### Fluorescence microscopy and imaging

All images were taken with a Nikon Upright Microscope Eclipse Ni-U using either a 40 (Nikon Plan Apo 40×/0.95, OFN25 DIC N2), 60 (Nikon Plan Apo 60×/0.95, OFN25 DIC N2), or 100× (Nikon Plan Apo 100×/1.45, OFN25 DIC N2) objective and a Nikon DS Ri2 camera. For image capture, the manual multichannel capture function of the NIS-Elements AR software (v. 4.30.02, 64-bit) was used. For images at 40 and 60× magnifications, DAPI and FITC channel (GFP, mNeonGreen) exposure times were 10–30 ms and 2 s, respectively. For 100× in oil immersion, exposure times were 300 ms (DAPI) and 4 s (FITC). The mean fluorescence intensity per inclusion was measured with the area analysis function by autodetecting the area of 30 individual inclusions per strain of interest.

### Statistics

GraphPad Prism (v. 10, GraphPad Software, Boston, MA, USA) was used for all statistical analyses. The Mann-Whitney test was used to compare two means of data that were not normally distributed, and an unpaired *t*-test was used if the data were normally distributed.

### Whole-genome sequencing and analysis

For long-read sequencing, libraries were prepared using the RBK114-24 Rapid Barcoding Kit and sequenced on a MinION device using R10.1.4 flow cells (Oxford Nanopore Technologies). Basecalling, adapter trimming, and demultiplexing were performed with Dorado (basecall server 7.3.9, SUP mode) implemented in MinKNOW 24.02.6 (Oxford Nanopore Technologies). Host sequences were removed by mapping reads to the *Macaca mulatta* genome (accession no. GCF_003339765.1) and collecting unmapped reads using minimap2 v2.24 (63) and samtools 1.15.1 (64). Following read filtering to a minimum length of 1,000 bp with nanoq v0.10.0 (65), assemblies were generated with Flye v2.9.3 (66).

## Data Availability

Genome assemblies are available under NCBI Bioproject No. PRJNA962280.

## SUPPLEMENTAL MATERIAL

Figures S1 and S2 spectrum.01641-25-s0001.pdf Fig. S1. Fluorescence intensity comparison between P787 and 1710S. Fig. S2. Vector map of pUC-Cabpl-GFP.

Table S1 spectrum.01641-25-s0002.xlsx All shuttle vectors used in this study either for *chlamydial* transformation or for vector construction.

Table S2 spectrum.01641-25-s0003.xlsx Table S2A. ompA confirmation. Table S2B. Stability assay. Table S2C. Overview transformation protocols.

Table S3 spectrum.01641-25-s0004.xlsx Contains all the Nanopore sequencing data provided in this study.

ASM does not own the copyrights to Supplemental Material that may be linked to, or accessed through, an article. The authors have granted ASM a non-exclusive, world-wide license to publish the Supplemental Material files. Please contact the corresponding author directly for reuse.
