# Supplementary material for: Development of shuttle vector-based transformation systems for veterinary and zoonotic chlamydiae
Source: Microbiol Spectr. 2025 Jul 23;13(9):e01641-25. doi: 10.1128/spectrum.01641-25 (PMC12403634; doi:10.1128/spectrum.01641-25)
Supplement: Figures S1 and S2 — Fig. S1. Fluorescence intensity comparison between P787 and 1710S. Fig. S2. Vector map of pUC-Cabpl-GFP. [file spectrum.01641-25-s0001.pdf]

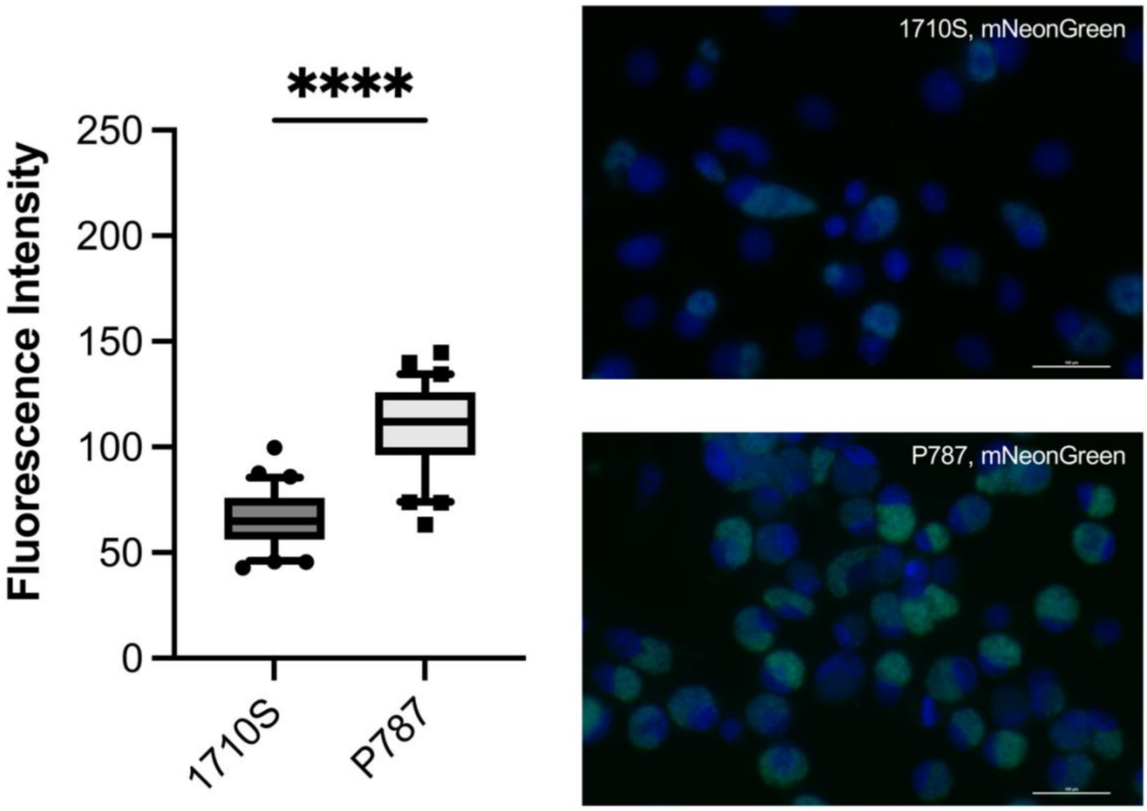

Figure S1. Shown is the mean fluorescence intensity of 30 inclusions of NeonGreen-tagged *C. pecorum* strain 1710S compared to P787. An unpaired t-test was used for statistical analysis. Four asterisks (****) represent p-values <0.0001. Representative images for each fluorophore were taken with a 40x objective (top right: GFP, bottom right: mNeonGreen). The white size bar represents 100 µm.

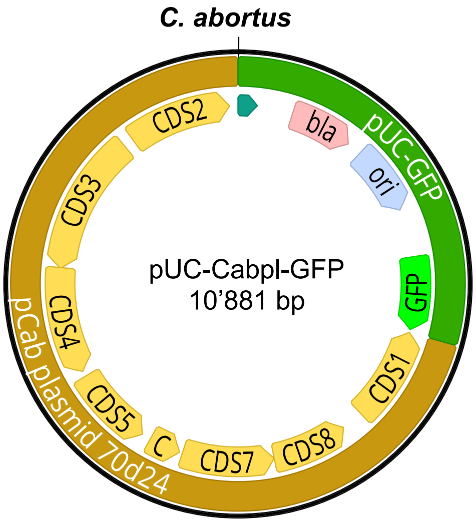

Figure S2. Shown is the *C. abortus* shuttle vector pUC-Cabpl-GFP comprising the native plasmid of *C. abortus* strain 15-70d24 as well as pUC-GFP, which contains a beta-lactamase (*bla,* pale red), the pUC origin of replication (*ori*, pale blue) and GFP (bright green). Created with Geneious version 2025.0 by Biomatters. Available from https://www.geneious.com.
